# Retraction: Hellinen et al. Drug Flux across RPE Cell Models: The Hunt for an Appropriate Outer Blood–Retinal Barrier Model for Use in Early Drug Discovery. *Pharmaceutics* 2020, *12*, 176

**DOI:** 10.3390/pharmaceutics14030595

**Published:** 2022-03-09

**Authors:** Laura Hellinen, Heidi Hongisto, Eva Ramsay, Kai Kaarniranta, Kati-Sisko Vellonen, Heli Skottman, Marika Ruponen

**Affiliations:** 1 School of Pharmacy, Faculty of Health Sciences, University of Eastern Finland, 70210 Kuopio, Finland; laura.pelkonen@uef.fi (L.H.); kati-sisko.vellonen@uef.fi (K.-S.V.); 2 Department of Ophthalmology, Institute of Clinical Medicine, University of Eastern Finland, 70210 Kuopio, Finland; heidi.m.hongisto@tuni.fi (H.H.); kai.kaarniranta@uef.fi (K.K.); 3 Drug Research Programme, Division of Pharmaceutical Biosciences, Faculty of Pharmacy, University of Helsinki, P.O. Box 56, FI-00014 Helsinki, Finland; eva.ramsay@helsinki.fi; 4 Department of Ophthalmology, Kuopio University Hospital, P.O.Box 100, FI-70029 Kuopio, Finland; 5 Faculty of Medicine and Health Technology, BioMediTech, Tampere University, 33520 Tampere, Finland; heli.skottman@tuni.fi

The published article [1] has been retracted due to data errors. During a review of the data, the authors found that the studied LEPI-1 cell line is not derived from the ARPE-19 RPE cell line of the human origin as described in the article but rather originates from the Madin-Darby canine (Canis familiaris) kidney cell line with the human MDR1 gene (MDCK-MDR1). This incorrect interpretation of the cell origin was a consequence of cell line cross-contamination and an error in characterization process.

The outcome and conclusions of the study were affected, so it was decided that a correction would not be appropriate, and the paper will therefore be retracted. We apologize for any inconvenience caused by the removal of this article.

To ensure the addition of only high quality scientific works to the field of scholarly publication, this article [1] is retracted and shall be marked accordingly. We apologize to the readers of *Pharmaceutics*.
